# Insulin pen use and diabetes treatment goals: A study from Iran STEPS 2016 survey

**DOI:** 10.1371/journal.pone.0221462

**Published:** 2019-08-28

**Authors:** Hedyeh Ebrahimi, Farhad Pishgar, Moein Yoosefi, Sedighe Moradi, Nazila Rezaei, Shirin Djalalinia, Mitra Modirian, Niloofar Peykari, Shohreh Naderimagham, Rosa Haghshenas, Saral Rahimi, Hamidreza Jamshidi, Alireza Esteghamati, Bagher Larijani, Farshad Farzadfar

**Affiliations:** 1 Non-Communicable Diseases Research Center, Endocrinology and Metabolism Population Sciences Institute, Tehran University of Medical Sciences, Tehran, Iran; 2 Department of Biostatistics, Faculty of Paramedical Sciences, Shahid Beheshti University of Medical Sciences, Tehran, Iran; 3 Endocrine Research Center, Institute of Endocrinology and Metabolism, Iran University of Medical Sciences, Tehran, Iran; 4 Deputy of Research and Technology, Ministry of Health and Medical Education, Tehran, Iran; 5 Ministry of Health and Medical Education, Tehran, Iran; 6 Pharmaceutical Sciences Branch, Islamic Azad University, Tehran, Iran; 7 School of Medicine, Department of Pharmacology, Shahid Beheshti University of Medical Sciences, Tehran, Iran; 8 Endocrinology and Metabolism Research Center, Vali-Asr Hospital, School of Medicine, Tehran University of Medical Sciences, Tehran, Iran; 9 Endocrinology and Metabolism Research Center, Endocrinology and Metabolism Clinical Sciences Institute, Tehran University of Medical Sciences, Tehran, Iran; Tabriz University of Medical Sciences, ISLAMIC REPUBLIC OF IRAN

## Abstract

**Background:**

Frequency of insulin pen use, despite its higher costs, is increasing to substitute the traditional use of insulin vials. This study aims to report insulin pen use frequency and its associated factors among participants of the STEPS survey 2016 in Iran, which was conducted based on the World Health Organization (WHO) STEPS methodology.

**Methods:**

In this cross-sectional study, 19,503 (mean age of 46.03±0.13) out of 30,541 participants of the Iran STEPS survey were included (Inclusion criteria: aged >25 years old and availability of their demographic, clinical, and laboratory results for serum glucose, HbA1c, and lipid profile). Clinical and demographic characteristics, a frequency of use of each diabetes mellitus treatment type, and the association of insulin pen use with health outcomes are reported using descriptive analysis and propensity score modeling.

**Results:**

There were 1,999(10.85%) individuals diagnosed with diabetes in the population, while 1,160(56.87%) cases were taking antihyperglycemic treatments. In this subset, 240(21.14%) individuals administered insulin with or without using oral agents at the same time. 52.28% of participants who were under insulin therapy used insulin pens. None of the socioeconomic determinants, including gender (p-value = 0.11), type of residential areas (p-value = 0.52), years of schooling (p-value = 0.27), wealth index (p-value = 0.19), marital status (p-value = 0.37), and insurance types (p-value = 0.72) were significantly different among groups using insulin pens and insulin vials. Moreover, in the propensity score modeling, pen usage was not associated with a lower heart attack and ischemic stroke histories, systolic blood pressure, serum lipid profile, blood glucose, or HbA1c levels.

**Conclusion:**

Results showed that the use of the higher-costing insulin pens compared to traditional vials and syringes is not associated with improved glycemic control and better lipid profile in our sample. Future studies are needed to confirm these findings and to compare other aspects of insulin pen use, including adherence to treatment and cost-effectiveness.

## Introduction

The increasing trends in prevalence and disability-adjusted life years (DALYs) attributed to diabetes are calling for actions to address the health and financial burdens of this disease. It has been shown that improving glycemic control with therapeutic interventions (ranging from lifestyle modification to oral antidiabetic medications and insulin injections) reduce diabetes mellitus complications.[1–3]

Insulin therapy is one of the most effective interventions to maintain glycemic control, which results in preventing the incidence and progression of diabetes complications.[4] However, there are several obstacles that lower frequency of use and adherence to insulin therapy, including needle phobia among patients, social stigma linked with insulin injection, errors in dosage adjustment, high costs of insulin, and injection pain.[5, 6] Insulin delivery method (using insulin pens or the traditional way of using vial and syringes) is another contributor to insulin usage in patients with diabetes. Multiple studies showed that insulin pen users take insulin more persistently and as a result, fewer hypoglycemic events compared to insulin vial users are reported in this group.[7–9] Moreover, Eby et al. reported that although in adults with type 2 diabetes insulin pens may impose the higher financial burden to healthcare systems than other insulin forms, insulin pen users have lower health-related costs, due to fewer hospitalizations and shorter length of stay.[10] However, the superior efficacy of insulin pens in maintaining glycemic control in patients with diabetes has been questioned in several studies.[11, 12]

Recent studies have reported an increasing trend in insulin pen use in developed countries.[13, 14] However, given the high burden of diabetes and the reported benefits in insulin pen application, data on the prevalence of insulin therapy in Iran are scarce. The present work is aimed to study insulin usage prevalence, frequency in use of insulin pens compared to insulin vials, and their associated factors, as well as to compare health outcomes in patients with diabetes taking insulin pen with insulin vial users, based on the nationally representative data of STEPS 2016 study in Iran.

## Methods

This study aims to estimate the frequency of insulin pen use among Iranian adults with diabetes and to study contributors to insulin pen usage based on data from the STEPS survey 2016.

### Study design and participants

In this study, we used data of a large-scale cross-sectional survey, STEPS 2016, which was nested within non-communicable disease (NCD) risk factor surveillance project. To run the STEPS 2016 survey in Iran, we used the suggested World Health Organization (WHO) STEPwise approach toward NCD risk factor surveillance (STEPS). Moreover, the WHO advised countries to modify risk factors and core variables of the study to meet the local and regional interests of them.[15] The full study protocol is presented elsewhere.[16] Briefly, Iranian residents (with Iranian nationality) aged more than 18 years (above 25 years old for lab tests), were surveyed in 3 phases. In phase 1, participants were asked about their sociodemographic characteristics, lifestyle data, medical histories, and history of known risk factors for NCD. Phase 2 and 3 of this study gathered information on anthropometric and laboratory tests, respectively.

The target population was selected through systematic cluster random sampling frame from rural and urban areas of 31 provinces of Iran, though later one province (Qom) declined to participate in the survey. For the selected subjects, a pre-designed digital questionnaire was completed as the first phase of the study. Out of the 31,050 subjects who were selected to enter the study, 30,541 completed the first phase. Following that, in the second phase, physical measurements of 30,042 of this sample were collected and were added to the database. However, only subjects aged more than 25 years were invited for the third phase, which consisted of serum and urine samples collection. 19,778 individuals participated in phase 3. Herein, we analyzed data of all participants, aged ≥25 years old and had available laboratory results for serum glucose, HbA1c, and lipid profile (19,503 subjects).

### Physical and biochemical measurements

Anthropometric measurements in this study included height (evaluated using a standard height ruler) and weight (measured in an upright position by a calibrated scale). Blood pressure of patients was measured in sitting position by digital devices (BM 20, Beurer, Germany) after 5 minutes of rest. This assessment was repeated two more times, each after 5 minutes of rest. The average of second and third assessments was used for interpretation.

Samples of venous blood were collected from eligible individuals following 12 hours of fasting. Using the autoanalyzer (Cobas C311 Hitachi, Tokyo, Japan), serum was assessed for levels of fasting plasma glucose (FPG), HbA1c, high-density lipoprotein cholesterol (HDL), total cholesterol, and triglycerides (TG). Low-density lipoprotein cholesterol (LDL) was calculated through Friedwald formula if the TG level was less than 400 mg/dL.[17] All the laboratory samples were transferred to a central laboratory to be assessed by one autoanalyzer to prevent inter-laboratory comparisons bias.

### Definitions

Diagnosis of diabetes was established based on subjects’ self-reports (being under treatments) or FPG levels of ≥126 mg/dl. Hypertension was defined as systolic blood pressure ≥140 mmHg or diastolic blood pressure ≥90 mmHg or use of antihypertensive medications. Body mass index (BMI) was calculated by dividing a person’s weight in kilograms by the square of height in meters. BMI was categorized into 4 classes, underweight (BMI less than 18.5), normal weight (BMI≥18.5 and <25), overweight (BMI≥25 and <30), and obese (BMI≥30 kg/m^2^). Wealth index (WI) was calculated by the Principal Component Analysis method (PCA), using the questionnaire data related to the home area, the number of rooms in the house, family assets, and home appliances that the family possessed. The calculated WI were categorized into its quintiles, where the first quintile showed the poorest income status and the fifth was the richest category. Years of schooling was the number of years which an individual had successfully finished and it was categorized into 4 subgroups (0, 1–6, 7–12, and >12 years of schooling).

To assess individuals’ physical activity, STEPS survey 2016 used the Global Physical Activity Questionnaire version 1 (GPAQ 1), developed by the World Health Organization (WHO). The GPAQ consists of 16 questions related to frequency, intensity, duration, and setting (transport-related, at work, and leisure time) of the respondent’s physical activity. Then, gathered data were converted to Metabolic Equivalent Tasks (METs), the ratio of an individual’s working metabolic rate compared to the resting metabolic rate that shows the intensity of physical activity. Moderate intensity activities and vigorous intensity activities were assigned a value of 4 and 8 METs, respectively. Finally, the total MET-min score was calculated through the sum of all MET-minutes per week.[18]

### Statistical analysis

To make our results representative for the whole population of Iran, we used three sampling weights related to each phase of the study in our analyses. These include the questionnaire, anthropometric, and laboratory weights. Each of these weights consisted of provincial, household, same age-sex group, specific non-response in each step, sampling, and individual non-response weights. We used these three weights in all of our analyses. Additionally, for the national estimates, we adjusted the weights for the population of Qom province, which was missing in our samples.

We used descriptive statistics to report the prevalence of diabetes and its awareness rate among our participants. Moreover, we showed and compared demographic and clinical characteristics of patients with diabetes between males and females, using t-tests and Chi-square tests. Several logistic regression models were built to assess roles of insulin pen use in serum levels of FPG, HbA1C, LDL, HDL, total cholesterol, and triglyceride, besides systolic blood pressure and histories of heart attacks and ischemic strokes in the past years. All models were controlled for effects of socioeconomic factors (types of insurance, the residential areas, years of schooling, wealth index, age, sex, and BMI). Then, propensity score modeling was used to assess causal relationships; we studied the contributing effects of using insulin pen on health outcomes (the aforementioned variables) in patients with diabetes. Additionally, by adopting this methodology, the possible problem with the non-random distribution of pen device use was addressed.[19, 20]

Throughout this work, quantitative and qualitative variables were shown as the mean ± standard error of the mean (SEM) and number (percentage), respectively. Statistical analyses were performed using Stata version 14 (Stata Corporation, College Station, TX, USA) and R software (R Foundation for Statistical Computing, Vienna, Austria), and propensity score modeling was conducted using MatchIt package (version 2.4–21) for R software. P-values less than 0.05 were considered as statistically significant.

### Ethical considerations

All participants were informed about the methods and goals of the study. Survey participation was voluntary and written informed consent forms were obtained from all of the participants. The final dataset was de-identified for analysis and the ethical approval for the study was obtained from the ethical committee of the National Institute for Medical Research Development (NIMAD) (ID: IR.NIMAD.REC.1394.032).

## Results

In this study, we included 19,503 subjects from 30 provinces of Iran (excluding Qom province), who were aged more than 25 years and had available laboratory results. Our study population consisted of 8,969(45.98%) men and 10,534(54.01%) women, with a mean age of 46.03±0.13 among both genders (47.18±0.19 and 46.70±0.18 for men and women, respectively; p-value = 0.07).

### Characteristics of patients

Using the mentioned criteria for diagnosis of diabetes, there were 1,999(10.85%) individuals with diabetes in our study population and the average age of adults with diabetes was 58.87±0.32, while the difference of mean age in men and women wasn’t statistically significant (59.16±0.49 and 58.66±0.42 in men and women, respectively; p-value = 0.44). A total 1,493(74.63%) subjects were aware of their disease status, among which 587 were men and 906 were women (70.33% of all men with diabetes and 77.74% of all females with diabetes; p-value = 0.01).

In this study and among the adult population with diabetes, there were 1,174 people (57.90% of all subjects, 441 men and 733 women) with hypertension, 104 people (4.91% of total, 64 men and 40 women) with heart attack in past year, and 41 people (1.88% of total, 19 men and 22 women) with stroke in past year. Moreover, our results showed that in our population there were 14 underweight people (0.66% of total, 10 men and 4 women), 376 people (20.43% of total, 200 men and 176 women) with normal weight, 774 overweight people (40.19% of total, 365 men and 409 women), and 760 people (38.72% of total, 224 men and 534 women) with obesity.

For the whole adult population with diabetes, means of HbA1c (%), LDL (mg/dL), HDL (mg/dL) and adjusted MET were 7.87±0.05 (7.85±0.08 in men and 7.89±0.06 in women; p-value = 0.73), 95.57±0.93 (93.38±1.59 in men and 97.11±1.14 in women; p-value = 0.06), 39.24±0.31 (35.93±0.42 in men and 41.65±0.41 in women; p-value = <0.001), and 1,302.02±72.02 (1,832.11±144.13 in men and 939.07±66.51 in women; p-value = <0.001), respectively.

### Treatment in patients with diabetes

The results showed that 1,160(56.87%) adult patients with diabetes were taking antihyperglycemic medical treatments, out of which 240(21.14%) individuals administered insulin with or without using oral agents concurrently. Moreover, 52.28% of patients who were under insulin therapy used insulin pens. Table 1 represents a complete overview of treatment coverage for diabetes in Iranian adult patients with diabetes.

**Table 1:** Treatment status and regimens among patients with diabetes.

	Total N (%)	Male N (%)	Female N (%)	*P-*value
No treatment	839 (43.13)	374 (46.89)	465 (40.42)	0.01
Receiving treatment	1,160 (56.87)	453 (53.11)	707 (59.58)	0.01
Oral antihyperglycemic agent	1,087 (78.86)	420 (81.09)	667 (77.43)	0.33
Insulin pen devices	136 (11.05)	54 (10.75)	82 (11.25)	
Insulin vial and syringes	104 (10.09)	33 (8.16)	71 (11.33)	

Categorical variables are shown as number (percentage). Chi-square test was used to compare variables between male and female, whenever applicable.

### Determinants of insulin pen administration

In order to compare socioeconomic determinants in patients using insulin pens and those using traditional vials and syringes, our results showed that none of the determinants, including gender (p-value = 0.11), type of the residential areas (p-value = 0.52), years of schooling categories (p-value = 0.27), WI quartiles (p-value = 19), marital status (p-value = 0.37), or insurance types (p-value = 0.72) were statistically significantly different among these two groups (Table 2).

**Table 2:** Association of patients’ demographic status and administration of insulin pen or vial.

Variables		Insulin vials N (%)	Insulin pens N (%)	*P*-value
Gender	Male	33 (30)	54 (40.11)	0.11
	Female	71 (70)	82 (59.89)	
Area type	Urban	84 (79.95)	106 (76.89)	0.52
	Rural	20 (20.05)	30 (23.11)	
Years of schooling	0 year	36 (35.16)	40 (30.12)	0.27
	1–6 year(s)	27 (24.27)	48 (34.51)	
	7–12 years	32 (30.65)	31 (22.72)	
	>12 years	9 (9.91)	17 (12.65)	
Wealth index	Quartile 1 (poorest)	10 (10)	18 (14.4)	0.19
	Quartile 2	34 (30.84)	28 (19.58)	
	Quartile 3	25 (25.62)	29 (22.27)	
	Quartile 4	21 (21.68)	31 (24.19)	
	Quartile 5 (richest)	12 (11.86)	27 (19.55)	
Marital status	Never married	0 (0)	1 (0.83)	0.37
	Married	80 (78.26)	116 (84.87)	
	Divorced	2 (1.89)	1 (0.69)	
	Widow	21 (19.85)	18 (13.62)	
Insurance Type	No Insurance	5 (4.5)	7 (5.5)	0.72
	Iran Health Insurance	28 (27.74)	48 (35.84)	
	Social Insurance	52 (50.35)	56 (40.64)	
	Army Insurance	8 (8.01)	9 (7.01)	
	Imam committee Insurance	1 (0.82)	2 (1.51)	
	Other Insurance	9 (8.59)	14 (9.49)	

Categorical variables are shown as number (percentage). Chi-square test was used to compare variables between males and females.

### Diabetes outcomes in patients using insulin pens

**Fig 1:**
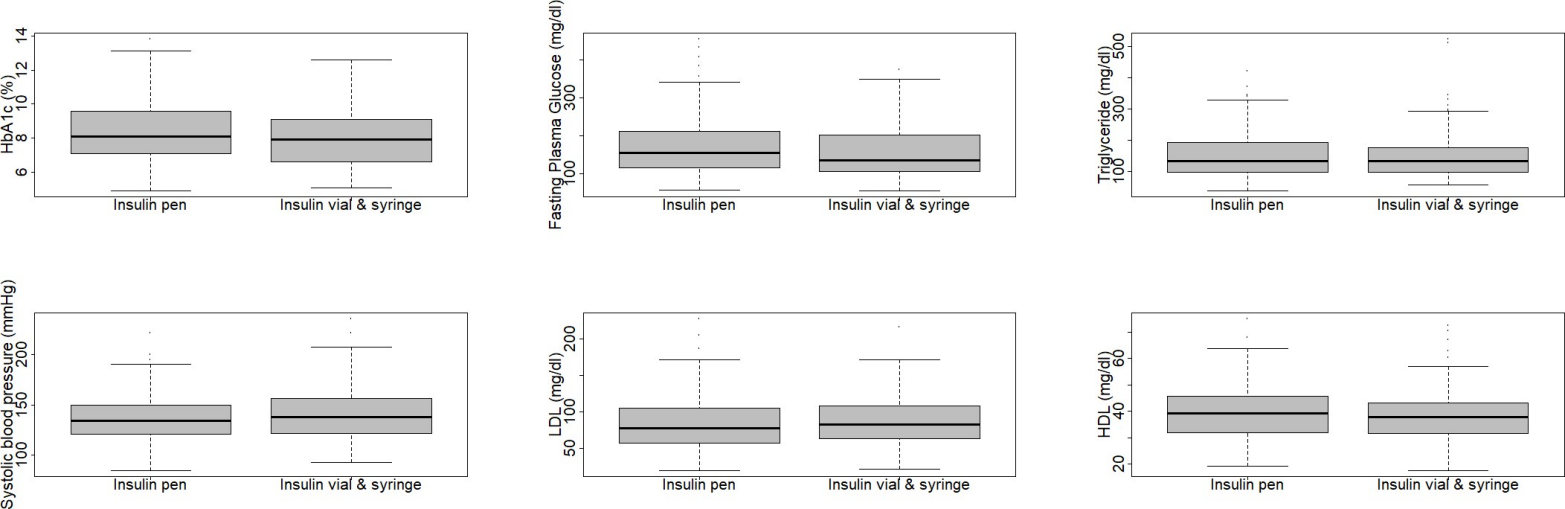
Comparison of health outcomes in patients using insulin pen and insulin vial. Serum levels of HbA1c (A), fasting plasma glucose (B), triglyceride (C), systolic blood pressure (D), low-density lipoprotein (E), and high-density lipoprotein (F) are shown in patients with diabetes using insulin pen and individuals using insulin vial, using box plot and whiskers.

We further studied levels of FPG, HbA1c, LDL, HDL, and systolic blood pressure in patients taking insulin pens and vials (Fig 1). We compared FPG, HbA1c, LDL, HDL, BMI, and systolic blood pressure among patients using insulin pen and insulin vial users, stratified for the gender of participants and their age groups (adults [30≤ age <60] and senior adults [≥60]). Our analyses showed comparable health outcomes in two groups of patients; no significant differences in serum levels of markers, systolic blood pressure, or BMI. We further built several logistic regression models to assess the association of insulin pen use with health outcomes (the FPG, HbA1C, LDL, HDL, total cholesterol, and triglyceride serum levels, in addition to systolic blood pressure and histories of heart attacks and ischemic strokes), adjusted for effects of different socioeconomic factors. No statistically significant association between insulin pen use and these health measures were shown in our study. Moreover, by adopting propensity score modeling (MatchIt), we assessed the effects of insulin delivery method on health outcomes in patients with diabetes treated with insulin. We showed that pen usage was not associated with health outcomes (Table 3).

**Table 3:** The estimated effect of insulin pen administration on health outcomes and comorbidities in patients with diabetes under insulin therapy (based on the MatchIt model).

Variable	Average effect (95% confidence interval)
FPG	9.74 (-10.01–29.89)
HbA1c	0.33 (-0.12–0.79)
LDL	-1.48 (-10.34–7.51)
HDL	1.23 (-1.41–3.91)
Total cholesterol	-1.46 (-13.39–10.81)
TG	-16.07 (-55.44–26.15)
Systolic blood pressure	-4.56 (-10.47–1.39)
Heart attack history	0.00 (-0.05–0.06)
Ischemic stroke history	0.01 (-0.02–0.06)

Low-density lipoprotein cholesterol, LDL; High-density lipoprotein cholesterol, HDL; Triglycerides, TG; BMI, Body mass index; Fasting plasma glucose, FPG

## Discussion

We conducted a cross-sectional study on 19,503 Iranian adults, urban or rural residents, to investigate the status of diabetes treatments in Iran and to assess roles of insulin pen devices in glycemic control improvement compared to insulin vials. Our results showed that among patient with diabetes who received insulin, 52.28% of cases used insulin pens, while there were no statistically significant differences in different demographic status (e.g. years of schooling, WI, marital status, insurance type, gender, and urban or rural resident area type) in subjects using insulin pens compared to insulin vials users. Clinical and laboratory outcomes also did not show statistically significant differences among patients with different insulin delivery forms.

Diabetes is the 5th cause with the highest DALYs among the non-communicable diseases in Iran, imposing 767,461 DALYs to the Iranian healthcare system only in 2016.[3] Considering the increasing burden of this disease, quality-essential interventions are required to both reduce the incidence of diabetes and also to improve the quality of diabetes treatments. Although the efficacy of regular insulin injection in developing and maintaining glycemic control has been highlighted in several reports, roles of insulin delivery forms in this regard are still a subject for debate.[2, 12]

Several studies reported that persistence and adherence to insulin therapy were higher in those subjects who used insulin pens and showed that both all-cause healthcare costs and annual treatment costs were lower in this group of patient with diabetes [7, 21–23] and reports on use of insulin pens among Indian and Lebanese populations showed that insulin pens are simpler, safer, and more convenient to use.[24, 25] However, there is a growing body of evidence showing that different insulin delivery forms are not associated with health-related outcomes among patients with diabetes; a systematic review and meta-analysis on the efficacy of pen devices and insulin vials showed that there were no significant differences in the number of patients achieving HbA1c treatment goal (<7%) among those who used insulin pens or vials (though the study reported improvements in the number of hypoglycemic events and mean but not clinically significant change of HbA1c in the group of adult patients treated with insulin pens).[12] Moreover, a multi-center study in the United States showed a negligible difference in glycemic control between patients taking prefilled, disposable pens and syringes.[26]

Furthermore, in a nationally representative trend analysis of the insulin delivery method in the United States, authors showed that between 2005 to 2011 among patients with type 2 diabetes, who initiated insulin therapy, frequency of administrating insulin pen for basal, mealtime, and mixtures analogs had increased dramatically, while this growth happened concurrently with a significant drop in initiating traditional insulin vials use.[13] Shahraz et al. used data of the National Health and Nutrition Examination Survey (NHANES) in the United States to investigate the health outcomes of patients with diabetes over time. Their results showed that over 2007 to 2014, the mean HbA1c level as an indicator of glycemic control in patients with diabetes did not change significantly.[27] Considering this result and the increased use of insulin pens in the United States over the similar time period, it can be concluded that despite a shift toward pen devices use among patients with diabetes, there were no improvements in glycemic control in this group of patients. In this study, we got similar results on the efficacy of insulin pen and we showed that use of pen device (compared to insulin vials use) do not improve glycemic control (in terms of fasting serum glucose and HbA1C levels). Moreover, our results were evident that lipid profile, hypertension, and risk of heart attack and ischemic stroke do not differ among groups with different methods of insulin delivery.

Javanbakht et al. in a prevalence-based cost of illness study in 2009, showed that patients diagnosed with type 2 diabetes consume approximately 9% of Iranian total health expenditure, while the average medical cost per capita was more than 840 USD in that year and the larger subcomponent of this measure is attributable to complication of diabetes and its medications.[28] Prefilled insulin pens are more expensive than insulin vials in developed, developing, and underdeveloped countries and the insulin products prices are rising over time.[29–31] In Iran, although insurance plans cover around 90% and 95% of the insulin pens and insulin vials costs, respectively, the price of insulin pen (adjusted to 1 mL 100 IU/mL), still is near six times the price of the insulin vials.[32, 33] Considering the above-mentioned facts and the background of Iranian economic inflation, we showed that health-related benefits (in terms of glycemic control and serum lipid profile impairments) in patients using insulin pens are insignificant.

We showed that the frequency of insulin pen and vials uses were not significantly different within groups with different socioeconomic characteristics. An explanation for this observation could be the improved access to basic health insurance coverage (covering insulin pen) after launching of health sector transformation plan in Iran, which may exacerbate the unnecessary healthcare costs in this regard.[34]

Although our results showed that insulin pens do not have superiority over insulin vials in controlling diabetes outcome in our sample, we cannot underestimate the patient preference for insulin pens. Considering the high cost of Insulin pens, future cost-effectiveness studies, which assess different types of insulin delivery, are needed in this field. Moreover, it is pivotal to consider the roles of Health Technology Assessment in policy-making, in order to recognize the proper model of insurance coverage for these drugs, given that currently the health transformation plan is being undertaken in Iran.[35]

Although we are reporting insulin pen use in a large sample of the Iranian population, our results may have been tempered by a couple of limitations. First, the Qom province denied to participate in Iran STEPS 2016 survey, and thus was dropped out from our analysis. We tried to address this shortcoming by calculating the non-response weight for Qom province and considering it in our analysis, which made our results representative of the national population. Moreover, the cross-sectional nature of this study limits its power to establish a causal association between glycemic control measures and the use of insulin pens in patients with diabetes. We highly recommend longitudinal studies on the role of insulin delivery methods assessing glycemic control among patients over time. Additionally, per STEPS questionnaire, data on the time period patients were taking the medications were not available for this study. Moreover, given the fact that currently in Iran, all insulin vials are human insulin and all insulin pens are analog, there may be a controversy surrounding the superiority of analogs over human insulin. However, the WHO Expert Committee on the Selection and Use of Essential Medicines concluded that there is no significant difference in the effectiveness of therapy between human and analog insulins. Furthermore, there is an inevitable chance of recall bias regarding the histories of heart attacks and ischemic strokes, and although we tried to minimize this bias by defining our survey question carefully, the potential impacts of this bias on our study findings cannot be fully addressed. Finally, in this study, although the majority of participants with diabetes had type 2 diabetes, we did not exclude patients with type 1 diabetes.

## Conclusion

This study is the first nationally representative study reporting patterns of insulin therapy, especially using pen devices and the associated health outcomes in an adult population with diabetes in Iran. Although there is a need to evaluate other aspects of insulin pen use, such as subjects’ preferences and adherence to therapy in cost-effectiveness and longitudinal studies, considering the increasing trend in diabetes prevalence and health charges attributed to this cause, our results showed that use of the higher-costing insulin pens is not associated with improved glycemic control, better lipid profile, or reduced heart attack and ischemic strokes in adult patients with diabetes in Iran.
